# Pharmacokinetic-Pharmacodynamic Evidence From a Phase 3 Trial to Support Flat-Dosing of Rifampicin for Tuberculosis

**DOI:** 10.1093/cid/ciae119

**Published:** 2024-03-11

**Authors:** Huy X Ngo, Ava Y Xu, Gustavo E Velásquez, Nan Zhang, Vincent K Chang, Ekaterina V Kurbatova, William C Whitworth, Erin Sizemore, Kia Bryant, Wendy Carr, Marc Weiner, Kelly E Dooley, Melissa Engle, Susan E Dorman, Payam Nahid, Susan Swindells, Richard E Chaisson, Pheona Nsubuga, Madeleine Lourens, Rodney Dawson, Radojka M Savic

**Affiliations:** Department of Bioengineering and Therapeutic Sciences, University of California, San Francisco, San Francisco, California, USA; Department of Bioengineering and Therapeutic Sciences, University of California, San Francisco, San Francisco, California, USA; Bakar Computational Health Sciences Institute, University of California, San Francisco, San Francisco, California, USA; UCSF Center for Tuberculosis, University of California, San Francisco, San Francisco, California, USA; Division of HIV, Infectious Diseases, and Global Medicine, University of California, San Francisco, San Francisco, California, USA; Department of Bioengineering and Therapeutic Sciences, University of California, San Francisco, San Francisco, California, USA; Department of Bioengineering and Therapeutic Sciences, University of California, San Francisco, San Francisco, California, USA; Centers for Disease Control and Prevention, Atlanta, Georgia, USA; Centers for Disease Control and Prevention, Atlanta, Georgia, USA; Centers for Disease Control and Prevention, Atlanta, Georgia, USA; Centers for Disease Control and Prevention, Atlanta, Georgia, USA; Centers for Disease Control and Prevention, Atlanta, Georgia, USA; University of Texas Health Science Center at San Antonio and the South Texas Veterans Health Care System, San Antonio, Texas, USA; Division of Infectious Diseases, Vanderbilt University Medical Center, Nashville, Tennessee, USA; University of Texas Health Science Center at San Antonio and the South Texas Veterans Health Care System, San Antonio, Texas, USA; Medical University of South Carolina, Charleston, South Carolina, USA; UCSF Center for Tuberculosis, University of California, San Francisco, San Francisco, California, USA; University of Nebraska Medical Center, Omaha, Nebraska, USA; Johns Hopkins University School of Medicine, Baltimore, Maryland, USA; Uganda-Case Western Reserve University Research Collaboration, Kampala, Uganda; TASK Applied Science CRS, Brooklyn Chest Hospital, Bellville, South Africa; Division of Pulmonology, Department of Medicine, University of Cape Town, Cape Town, South Africa; Department of Bioengineering and Therapeutic Sciences, University of California, San Francisco, San Francisco, California, USA; UCSF Center for Tuberculosis, University of California, San Francisco, San Francisco, California, USA

**Keywords:** tuberculosis, rifampicin, population pharmacokinetics, flat-dosing, weight-banded dosing

## Abstract

**Background:**

The optimal dosing strategy for rifampicin in treating drug-susceptible tuberculosis (TB) is still highly debated. In the phase 3 clinical trial Study 31/ACTG 5349 (NCT02410772), all participants in the control regimen arm received 600 mg rifampicin daily as a flat dose. Here, we evaluated relationships between rifampicin exposure and efficacy and safety outcomes.

**Methods:**

We analyzed rifampicin concentration time profiles using population nonlinear mixed-effects models. We compared simulated rifampicin exposure from flat- and weight-banded dosing. We evaluated the effect of rifampicin exposure on stable culture conversion at 6 months; TB-related unfavorable outcomes at 9, 12, and 18 months using Cox proportional hazard models; and all trial-defined safety outcomes using logistic regression.

**Results:**

Our model-derived rifampicin exposure ranged from 4.57 mg · h/L to 140.0 mg · h/L with a median of 41.8 mg · h/L. Pharmacokinetic simulations demonstrated that flat-dosed rifampicin provided exposure coverage similar to the weight-banded dose. Exposure-efficacy analysis (n = 680) showed that participants with rifampicin exposure below the median experienced similar hazards of stable culture conversion and TB-related unfavorable outcomes compared with those with exposure above the median. Exposure-safety analysis (n = 722) showed that increased rifampicin exposure was not associated with increased grade 3 or higher adverse events or serious adverse events.

**Conclusions:**

Flat-dosing of rifampicin at 600 mg daily may be a reasonable alternative to the incumbent weight-banded dosing strategy for the standard-of-care 6-month regimen. Future research should assess the optimal dosing strategy for rifampicin, at doses higher than the current recommendation.

## Teaser

Flat-dosing of rifampicin at 600 mg daily provided similar plasma exposure to conventional weight-banded dosing for drug-susceptible tuberculosis. This dosing strategy may be a reasonable alternative to weight-banded dosing in the 6-month standard-of-care regimen for drug-susceptible tuberculosis.

Rifampicin is the cornerstone drug in the current standard-of-care treatment for drug-susceptible tuberculosis (TB) due to its activity against replicating *Mycobacterium tuberculosis* (*Mtb*) and against persister, nonreplicating bacilli [1]. However, current rifampicin dosing is implemented based on outdated knowledge and has remained suboptimal [2]. The weight-banded dosing strategy recommends taking rifampicin at 8–12 mg/kg per day with a maximum daily dose of 600 mg [3, 4]. Adjusting the dose based on weight bands could be complicated as patients experience fluctuations in body weight during the treatment period [5, 6]. A pharmacokinetic (PK) analysis based on the phase 2A study of high-dose rifampicin suggested that rifampicin can be given as a flat dose and found that weight-banded dosing did not achieve a clinically relevant decrease in exposure variability compared with flat-dosing [5]. Weight-banded dosing may also complicate the conduct of clinical trials; a phase 2B dose-ranging trial of high-dose rifampicin found that, while weight was balanced across 3 randomized arms, weight-banded dosing led to an unexpected distribution of allocated rifampicin dose [6].

Understanding the connection between rifampicin's PK and treatment outcomes is critical in enhancing the current standard treatment for drug-susceptible TB [7]. Steady-state area under the concentration time curve (AUC_ss_) has been regarded as a useful surrogate drug-exposure marker to predict clinical outcomes [8]. Rifampicin is well tolerated in the standard regimen [9–11]. However, due to highly variable PK and a short half-life, suboptimal concentrations have been associated with decreased 2-month sputum culture conversion and increased treatment failure and relapse rates [6, 12–14]. Further examination of relationships between rifampicin exposure and long-term clinical outcomes can provide additional evidence for recommending a specific dosing strategy.

The Tuberculosis Trials Consortium (TBTC) with the AIDS Clinical Trials Group (ACTG) has conducted Study 31/ACTG A5349 (S31/A5349), a landmark phase 3 trial that demonstrated noninferiority of a 4-month rifapentine-moxifloxacin–containing regimen compared with the standard 6-month control regimen [15, 16]. Since all participants received 600 mg daily rifampicin as a flat dose in the control regimen, we leveraged this robust dataset to assess the flat-dosing strategy from PK, efficacy, and safety perspectives. Here, we aimed to (1) compare simulated rifampicin exposures achieved by flat-dosing and weight-banded dosing, (2) evaluate relationships between rifampicin exposure with clinical efficacy and safety outcomes, and (3) recommend a rifampicin dosing strategy.

## METHODS

### Study Design and Pharmacokinetic Sampling

This analysis was based on data collected from S31/A5349 (NCT02410772), a phase 3, international, multicenter, randomized, open-label, noninferiority trial. Participants were 12 years of age or older with drug-susceptible pulmonary TB. The participants were randomly assigned in a 1:1:1 ratio to 1 of 3 regimens: a standard 6-month TB regimen and two 4-month rifapentine-containing regimens. Rifampicin was administered at a 600-mg once-daily dose without food in the control regimen. In the initial 2 months, rifampicin was given along with isoniazid, pyrazinamide, and ethambutol. In the following 4 months, rifampicin was given only with isoniazid. Plasma PK samples were collected at approximately 0.5, 5.5, and 15.5 hours after the dose during weeks 2 to 8 and measured by high-performance liquid chromatography coupled to tandem mass spectrometry. Detailed methods have been published previously [15, 16].

### Modeling Software and Methods

We performed data management, analysis, and visualization with R (version 3.6.1) [17]. We analyzed PK data using the nonlinear mixed-effects modeling software NONMEM (version 7.5.1, Icon Development Solutions, Dublin, Ireland) and Perl-speaks-NONMEM (version 5.3.0). We estimated population PK parameters with stochastic approximation expectation-maximization with Laplacian estimation and the importance sampling method. The lower limit of quantification (LLOQ) for rifampicin was 0.1 mg/L. We censored rifampicin concentrations below the limit of quantification (BLQ) and modeled them using the M3 method [18].

### Model-Building Procedures

We divided the PK dataset into an analysis dataset (two-thirds) for model development and a validation dataset (one-third) for model validation, as detailed in Table 1. We considered different absorption models, including lag time and transit compartments; we also considered different residual error models (additive, proportional, and combination). We selected covariate effects using a stepwise procedure with forward inclusion (*P* < .05) and backward elimination (*P* < .01). We considered the following covariates: sex, race, age, body weight, body mass index (BMI), smear grade, Karnofsky score, cavitation on chest radiograph, extent of disease on chest radiograph, and people with human immunodeficiency virus (HIV) and diabetes. We considered several methods for allometric scaling of apparent clearance (CL/F) and volume of distribution (V/F) using body weight and fat-free mass, as previously described by Svensson et al [19]. We included covariates in the final model based on statistical significance, biological plausibility, and clinical relevance.

**Table 1.** Participant Characteristics

	Analysis Cohort (n = 490)	Validation Cohort (n = 232)	Full Cohort (n = 722)
Demographics			
** **Age, median (range), y	30 (13–77)	31 (14–63)	31 (13–77)
Male sex	346 (71)	165 (71)	511 (71)
Height, median (range), cm	167 (140–194)	168 (143–200)	167 (140–200)
Weight, median (range), kg	53 (40–122)	53.5 (40–98)	53 (40–122)
BMI, median (range), kg/m^2^	19.0 (12.8–45.4)	18.9 (13.7–33.8)	18.9 (12.8–45.4)
Race			
Black	348 (71)	166 (72)	514 (71)
Asian	59 (12)	29 (13)	88 (12)
Mixed/multiracial	74 (15)	34 (15)	108 (15)
White	9 (2)	3 (1)	12 (2)
Sub-Saharan African site	358 (73)	178 (77)	536 (74)
Clinical factors			
Cavitation on chest radiograph^a^			
Absent	125 (26)	53 (23)	178 (25)
<4 cm	158 (32)	84 (36)	242 (34)
≥4 cm	206 (42)	92 (40)	298 (41)
Extent of disease on chest radiograph^a^			
** **Lesions less than one-fourth of thoracic area	74 (15)	37 (16)	111 (15)
Lesions one-fourth to less than one-half of thoracic area	218 (45)	99 (43)	317 (44)
Lesions one-half or more of thoracic area	197 (40)	93 (40)	290 (40)
WHO smear grade^b^			
Negative	16 (3)	6 (3)	22 (3)
Scanty or 1–9 acid-fast bacilli	80 (16)	39 (17)	119 (17)
1+	107 (22)	68 (29)	175 (24)
2+	157 (32)	62 (27)	219 (30)
3+	129 (26)	57 (25)	186 (26)
Karnofsky score, median (range)	90 (70–100)	90 (60–100)	90 (60–100)
Living with HIV^c^	38 (8)	16 (7)	54 (7)
Living with diabetes	16 (3)	11 (5)	27 (4)
Evaluable PK samples			
Total evaluable	1427	681	2108
Below limit of quantification	333 (23)	147 (22)	480 (23)

Values are shown as n (%) unless otherwise specified. The entire PK dataset was split into model analysis and validation cohorts. The split was performed by randomly stratifying participants based on clinical site and HIV status, which aligns with the original trial design.

Abbreviations: ANOVA, analysis of variance; BMI, body mass index; HIV, human immunodeficiency virus; PK, pharmacokinetics; WHO, World Health Organization.

^a^Four participants did not have chest radiograph results available.

^b^Significant proportion test for binary categorical variables and ANOVA for more than 2 categorical variables (*P* < .05) when comparing model development and validation cohorts. One participant had unknown smear grade.

^c^One participant had unknown HIV status.

### Pharmacokinetic Simulations of Flat- and Weight-Banded Dosing

We conducted simulations to evaluate the impact of dosing strategies on rifampicin exposure. Specifically, we compared 600 mg daily rifampicin given at a flat dose with the daily weight-banded dose (450 mg for patients weighing 40–54 kg and 600 mg for those weighing ≥55 kg) using a virtually constructed cohort. We built this virtual population by combining available data from S31/A5349 and demographic characteristics from a pooled dataset that encompasses 4 other modern clinical studies, including OFLOTUB (NCT00216385), REMoxTB (NCT00864383), and RIFAQUIN (ISRCTN44153044) [20]. We restricted the range of possible body weights to 40 to 122 kg, consistent with the S31/A5349 trial population. The combined dataset comprised a total of 5687 participants; we performed 500 Monte Carlo simulations per participant for each dosing strategy.

### Pharmacokinetic-Efficacy and Pharmacokinetic-Safety Analysis

We investigated the relationships between rifampicin exposure and clinical outcomes using individual model-predicted AUC_ss_. We used time to TB-related unfavorable outcomes as our primary efficacy outcome due to its close representation of treatment response. A TB-related unfavorable outcome was defined in S31/A5349 as 2 consecutive positive cultures on or after week 17, not seen at month 12 with the last culture positive, or clinical diagnosis of TB recurrence and treatment reinitiation. The secondary efficacy outcome included time to stable culture conversion in the Mycobacteria Growth Indicator Tube (MGIT) assay (Beckton Dickinson, Maryland, USA). The efficacy population comprised all participants who underwent randomization in the microbiologically eligible (aka modified intention-to-treat) population, who were allocated to the standard 6-month control regimen. We evaluated the contribution of rifampicin exposure to efficacy outcomes using Cox proportional hazards models adjusted for Xpert MTB/RIF (Cepheid, Sunnyvale, California, USA) cycle threshold, extent of disease on chest radiograph, site (African vs non-African site), and people with HIV with the R survival package (version 3.5.5) [21]. We dichotomized rifampicin exposure by the median in the main analysis, and by continuous 5 mg · h/L decrements as sensitivity analysis.

The primary safety outcome was any grade 3 or higher adverse event (AE) during the on-treatment period, referring to the time during which the trial participants were receiving study medications and up to 14 days after the last dose. The secondary safety outcome was any treatment-related grade 3 or higher AE. Additional safety outcomes included serum total bilirubin 3 or more times over the upper limit of normal (ULN); alanine transaminase (ALT) and aspartate transaminase (AST) levels 5 and 10 times over the ULN, respectively; serious AEs, Hy's law; death; and premature discontinuation of the assigned regimen for any AE or for any reason other than microbiological ineligibility. The safety population included all randomized participants who started study treatment (aka modified intention-to-treat). We performed logistic regression with the R stats package (version 4.2.2). We first considered rifampicin AUC_ss_ in univariable logistic regression with respect to each of the safety outcomes mentioned above. We selected covariates for the final multivariable model using a stepwise procedure with forward inclusion (*P* < .10) and backward elimination (*P* < .05) based on the likelihood ratio test. All tests were 2-sided and *P* values <.05 were considered statistically significant.

## RESULTS

### Pharmacokinetic Model

**Figure 1.**
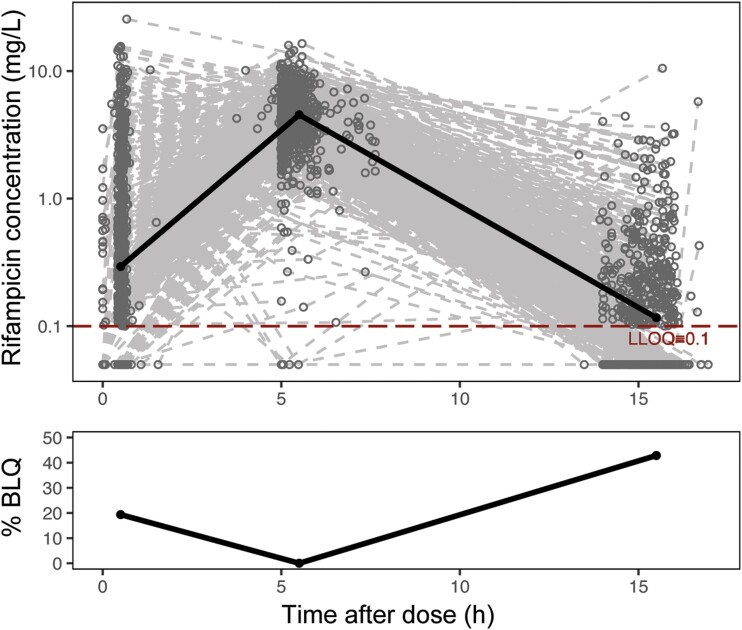
Observed rifampicin concentration over time after dose. In the top panel, the population's median plasma concentration trajectory is shown as a solid line, with LLOQ at 0.1 mg/L (dashed line). In the bottom panel, the median percentage of samples BLQ are shown as a solid line. The following bins were used to calculate the median values: 0.5 to 3, 3–8, and 8–20 hours. Abbreviations: BLQ, below the limit of quantification; LLOQ, lower limit of quantification.

**Figure 2.**
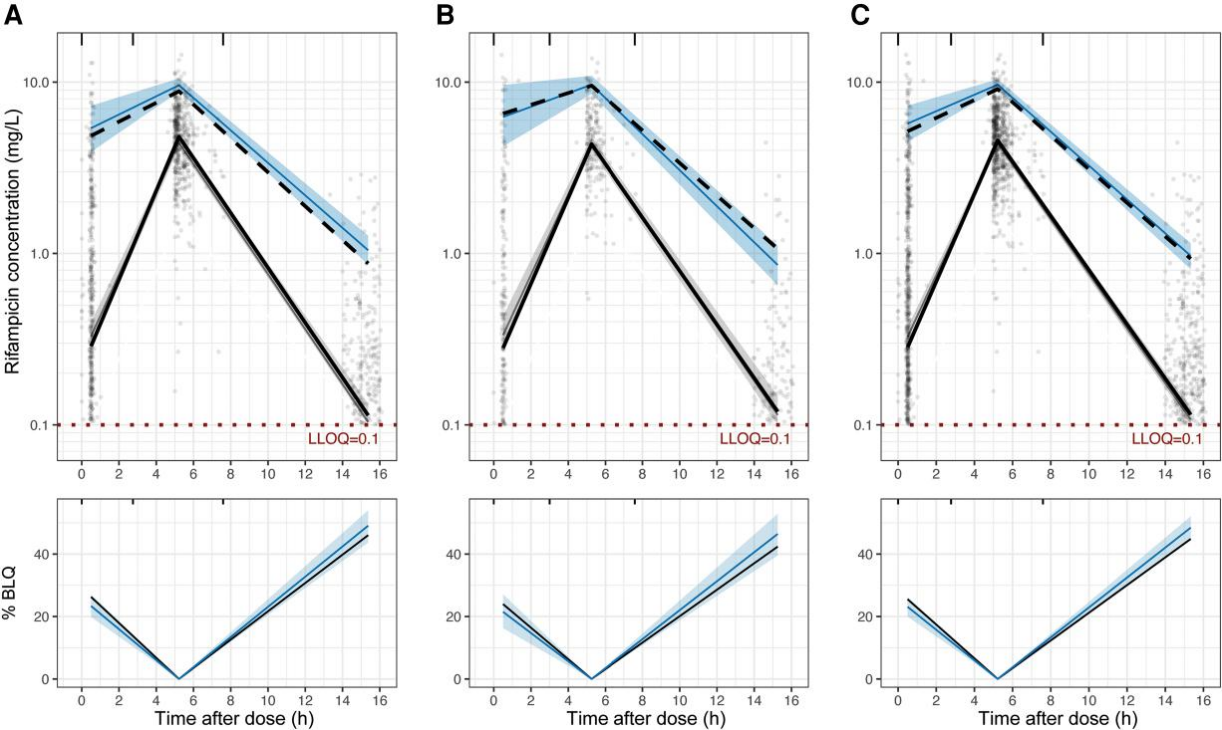
Visual predictive check of the final population PK model in the analysis cohort (*A*), validation cohort (*B*), and full cohort (*C*). In the top panels, model predictions (gray-shaded area [median] and blue-shaded area [95th percentile]) are shown overlaid with observations (solid black line [median] and dashed black line [95th percentile]) with LLOQ at 0.1 mg/L. Dots represent individual observed rifampicin concentrations. In the bottom panels, model-predicted median percentages of samples BLQ (blue shaded area) are shown overlaid with observations (black solid line). The following bins were used to calculate the summary statistics: 0.5–3, 3–8, and 8–20 hours. The observed fifth-percentile data were removed due to high percentages of BLQ at 0.5 and 15.5 hours after dose. Abbreviations: BLQ, below the limit of quantification; LLOQ, lower limit of quantification; PK, pharmacokinetics.

A total of 722 participants in S31/A5349 had rifampicin PK data available and were included in the analysis. Participant demographics and clinical factors used for model building and validation are summarized in Table 1. The final dataset included 2108 evaluable PK samples with 480 (23%) BLQ samples. Profiles of rifampicin plasma concentrations over time are shown in Figure 1. Note that PK sampling collection did not capture the expected peak concentration (C_max_) around 2 hours after dose [22] and resulted in high percentages of BLQ samples at approximately 5.5 and 15.5 hours after dose. A 1-compartment model with linear elimination and 4 transit compartments best described our data (Supplementary Figure 1). The final PK model parameters are shown in Table 2. Standard allometric scaling was selected to model the effects of body weight on CL/F and V/F due to superior statistical significance and model fits. In a typical participant with a 53-kg body weight, the mean transit time, CL/F, and V/F were 2.06 hours, 19.6 L/h, and 49.3 L, respectively. Our model agrees well with the observed data where the observed data overlaid on the shaded model-predicted areas, as shown in Figure 2.

**Table 2.** Final Parameter Estimates for Rifampicin Population PK Model

Parameter	Estimate	RSE, %
CL/F,^a^ L/h	19.6	2.01
V/F,^a^ L	49.3	2.62
MTT,^b^ h	2.06	2.36
Covariate effects on CL/F, %		
Asian relative to Black/mixed race	−10.1	21.9
Female relative to male	−22.9	15.5
Covariate effects on V/F, %		
Female relative to male	−20.7	17.2
Interindividual variability,^c^ %		
IIV CL/F	44.8	7.10
IIV V/F	32.1	25.6
IIV MTT	57.4	3.50
Correlation,^c^ %		
CL/F-V/F	50.5	3.49
CL/F-MTT	76.9	2.18
V/F-MTT	57.5	2.23
ε_prop_ in log scale	0.256	18.7

Abbreviations: CL/F, apparent clearance; CV, coefficient of variation; IIV, interindividual variability; MTT, mean transit time; PK, pharmacokinetics; RSE, relative standard error; V/F, apparent volume of distribution; ε_prop_, proportional error.

^a^The parameters CL/F and V/F were allometrically scaled using body weight (WT) and were described by: CL-WT cov = (WT/70)^0.75^; V-WT cov = (WT/70)^1^.

^b^The number of transit compartments is 4.

^c^IIV CL/F, IIV V/F, correlation CL/F-V/F, correlation CL/F-MTT, and correlation V/F-MTT are described as CV%.

### Impact of Covariates on Pharmacokinetics

**Figure 3.**
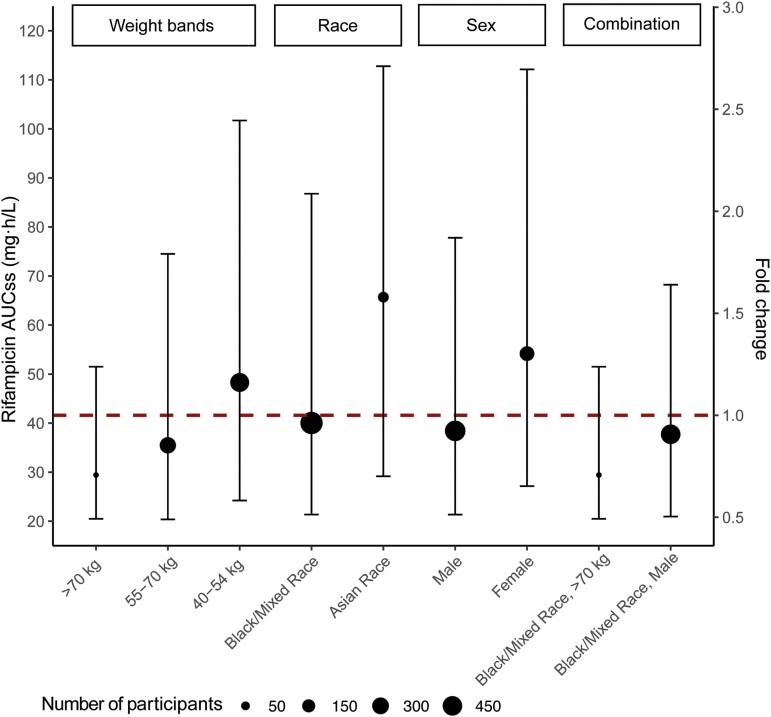
Model-derived rifampicin exposure stratified by significant covariates (body weight, sex, and race). Dots and bars represent stratified medians and ranges (5th to 95th percentiles) for each subpopulation. The size of dots shown corresponds to the number of participants. The dashed horizontal line corresponds to median exposure of entire population or fold change of 1 (41.8 mg·h/L). Abbreviation: AUC_ss_, area under the plasma concentration-time curve at steady state.

Sex, race, and body weight were the most important covariates contributing to variability in CL/F and V/F (Figure 3). Our final model indicated that the median AUC_ss_ of male participants, 38.4 (90% confidence interval [CI]: 21.4–77.8) mg · h/L, was approximately 30% lower than that of female participants, 54.2 (90% CI: 27.2–112) mg · h/L. The median AUC_ss_ of Black or mixed-race participants, 40.0 (90% CI: 21.4–86.7) mg · h/L, was approximately 40% lower than that of Asian participants, 65.7 (90% CI: 29.2–113) mg · h/L. The median AUC_ss_ of participants who weighed 40–54 kg, 55–70 kg, and more than 70 kg were 48.3 (90% CI: 24.2–102), 35.2 (90% CI: 19.9–69.4), and 33.0 (90% CI: 22.3–53.4) mg · h/L, respectively.

### Pharmacokinetic Simulations of Flat- and Weight-Banded Dosing

**Figure 4.**
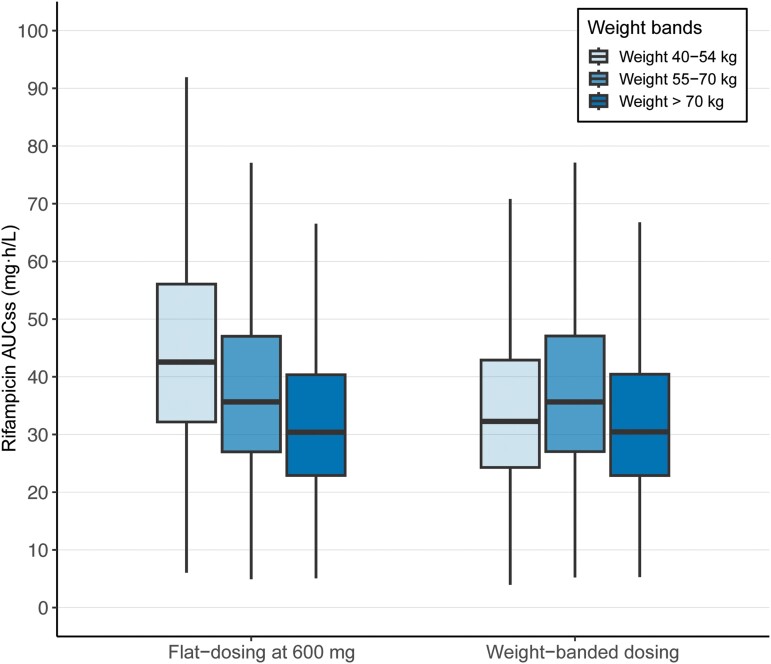
Comparable exposure from 600 mg rifampicin given as flat-dosing and weight-banded dosing. A total of 500 simulations were performed using a virtual population of persons with TB (N = 5687) consisting of demographic factors from 4 trials: S31/A5349 (NCT02410772), OFLOTUB (NCT00216385), REMoxTB (NCT00864383), and RIFAQUIN (ISRCTN44153044). In the standard 6-month control regimen, the flat-dosing strategy means administering rifampicin at 600 mg daily, while weight-banded dosing strategy means administering rifampicin daily based on weight bands (450 mg for patients weighing 40–50 kg and 600 mg for those weighing ≥55 kg). Abbreviations: AUC_ss_ area under the plasma concentration-time curve at steady state; TB, tuberculosis.

In our simulations shown in Figure 4, the flat-dosing strategy generated median AUC_ss_ values of 43.1 (90% CI: 21.6–87.1), 35.8 (90% CI: 18.1–71.1), and 30.4 (90% CI: 15.2–61.4) mg · h/L for the 40–54-, 55–70-, and >70-kg weight bands, respectively. On the other hand, the weight-banded dosing strategy led to median AUC_ss_ values of 32.3 (90% CI: 16.2–65.3), 35.8 (90% CI: 18.2–70.9), and 30.5 (90% CI: 15.2–61.2) mg · h/L for the 40–54-, 55–70-, and >70-kg weight bands, respectively.

### Pharmacokinetic-Efficacy and Pharmacokinetic-Safety Analysis

**Figure 5.**
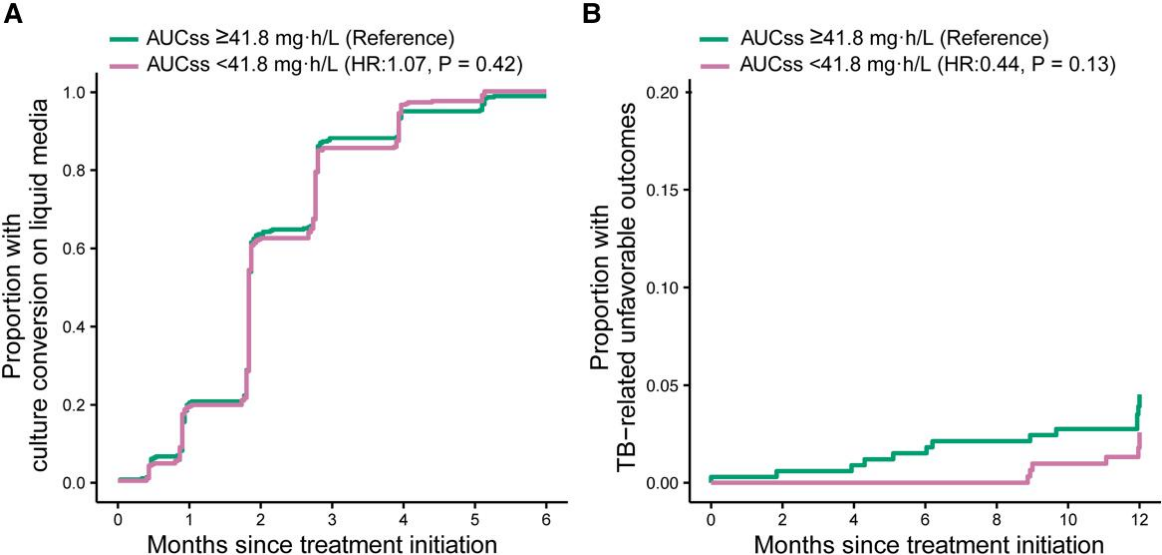
Cumulative event curves for (*A*) time to stable culture conversion at 6 months and (*B*) time to TB-related unfavorable outcomes at 12 months stratified by median exposure of rifampicin. Hazard ratios and *P* values were adjusted for HIV status, Xpert MTB/RIF cycle threshold, extent of disease on chest radiograph, and site (African vs non-African site). Abbreviations: AUC_ss_, area under the plasma concentration-time curve at steady state; HIV, human immunodeficiency virus; HR, hazard ratio; TB, tuberculosis.

All participants with PK data (N = 722) were included in the safety analysis and 680 from the microbiologically eligible population were included in efficacy and tolerability analyses. Dichotomization of participants with rifampicin AUC_ss_ below the median in comparison to the group above the median showed that there was no evidence that the 2 groups differed with respect to stable culture conversion at 6 months after treatment initiation and TB-related unfavorable outcomes (at 9, 12, and 18 months after treatment initiation) (Table 3 and Figure 5). In our sensitivity analysis, a 5-mg·h/L decrease in rifampicin AUC_ss_ was also not associated with efficacy endpoints (data not shown).

**Table 3.** Hazard of Stable Culture Conversion and Tuberculosis-Related Unfavorable Outcome by Rifampicin Exposure

Time Interval^a^	Rifampicin AUC_ss_ Below Median, Estimate (95% CI)	*P* ^b^
Hazard ratio for time to stable culture conversion		
6-Month	1.07 (.91–1.25)	.42
Hazard ratio for time to TB-related unfavorable outcome		
9-Month	.40 (.08–2.11)	.28
12-Month	.44 (.15–1.26)	.13
18-Month	.73 (.29–1.82)	.50

The exposure of interest was rifampicin AUC_ss_ below the median of 41.8 mg·h/L in comparison to at or above the median of 41.8 mg·h/L (reference group).

Abbreviations: AUC_ss_, area under the plasma concentration-time curve at steady state; CI, confidence interval; HIV, human immunodeficiency virus; TB, tuberculosis.

^a^Time interval was measured from the date of randomization.

^b^Each statistical test was adjusted for HIV status, Xpert MTB/RIF cycle threshold, extent of disease on chest radiograph, and site (African vs non-African site).

**Figure 6.**
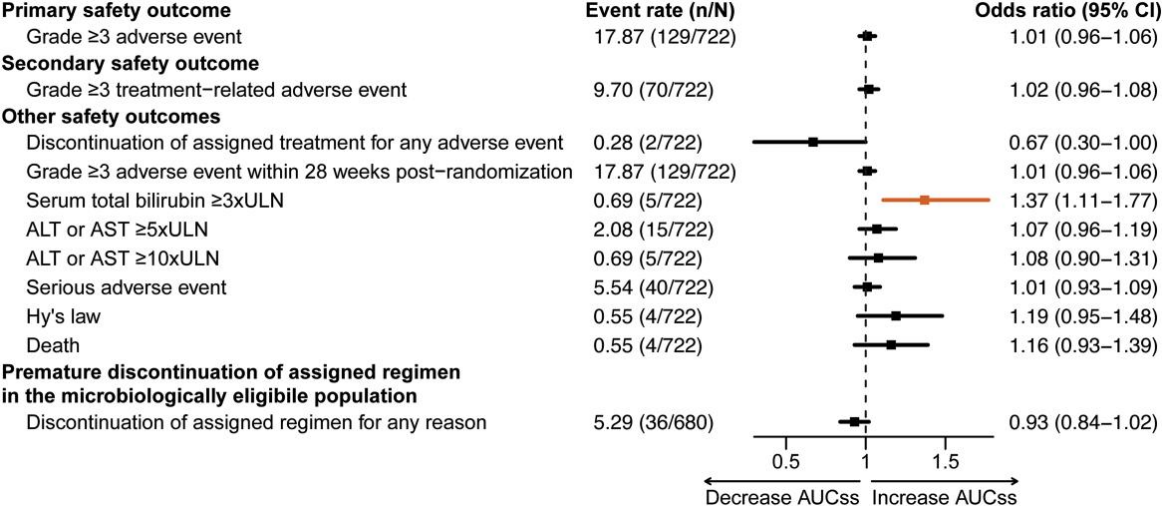
Odds of safety outcomes by rifampicin exposure. Odds ratios were calculated per 5-mg·h/L increase in rifampicin AUC_ss_ after adjustment for pyrazinamide AUC_ss_, age, and body weight. The safety outcome is highlighted in orange if it was found to be statistically significant with a *P* value <.05. Safety and tolerability outcomes were defined as those occurring during treatment and up to 14 days after discontinuation of study drug. Abbreviations: ALT, alanine aminotransferase; AST, aspartate aminotransferase; AUC_ss_, area under the plasma concentration-time curve at steady state; CI, confidence interval; n/N, number of participants reported for each safety outcome over of the total number of participants analyzed; ULN, upper limit of normal.

In univariable analysis (Supplementary Table 1), a 5-mg·h/L increase in rifampicin AUC_ss_ was associated with 4 safety outcomes: ALT or AST ≥5 × ULN (unadjusted odds ratio [OR], 1.16; 95% CI: 1.07–1.26), ALT or AST ≥10 × ULN (unadjusted OR, 1.24; 95% CI: 1.09–1.43), serum total bilirubin ≥3 × ULN (unadjusted OR, 1.36; 95% CI: 1.18–1.64), and Hy's law (unadjusted OR, 1.25; 95% CI: 1.07–1.46). In our multivariable analysis adjusted for pyrazinamide AUC_ss_, age, and body weight, a 5-mg·h/L increase in rifampicin AUC_ss_ was significantly associated with serum total bilirubin ≥3 × ULN (adjusted OR, 1.37; 95% CI: 1.11–1.77) (Figure 6, Supplementary Table 2).

## DISCUSSION

We leveraged the large S31/A5349 trial population, where participants allocated to the control regimen received a single dose of 600 mg rifampicin daily, to report the PK, efficacy, and safety of flat-dosed rifampicin and found that it was an adequate alternative to the longstanding practice of weight-banded dosing strategy. Flat-dosed rifampicin was best explained by a population PK model with a simple 1-compartment model with transit absorption and linear clearance. This structural model and parameter estimation are consistent with the rifampicin model widely utilized in previous PK models [19, 23–26]. Model-predicted AUC_ss_ was found to be comparable to that from previous studies and, thus, was used for the exposure metric of choice [5, 27].

Male sex, higher body weight, and Black/mixed race were associated with lower rifampicin exposure. The exposure fold reductions in these 3 subpopulations were modest at less than 0.75-fold change from the population median. We found large interindividual variability in rifampicin exposure across all subpopulations, which was expected of rifampicin PK [27]. In the absence of a randomized head-to-head comparison between flat-dosed and weight-banded dosed arms, we compared dosing strategies based on simulations in a virtual TB population. For the 40–54-kg weight band, flat-dosing showed a slightly higher median AUC_ss_ compared with weight-banded dosing (43.1 vs 32.3 mg·h/L, respectively). The overall exposure coverage of the 2 dosing strategies largely overlapped. Even with weight-banded dosing, rifampicin exposure remained highly variable. From the PK perspective, in agreement with Susanto et al [5], our simulations suggested that weight-banded dosing would provide only marginal benefits.

Exposure-efficacy analysis revealed that lower rifampicin exposure did not affect the hazard of stable culture conversion or TB-related unfavorable outcomes. The lack of association was likely attributed to the high response rate and strong adherence to the standard 6-month regimen in the S31/A5349 trial. Fewer than 5% of participants experienced any TB-related unfavorable outcomes 12 months after randomization, attenuating our ability to detect any potential relationship despite the large sample size. Moreover, robust adherence to the regimen possibly played a positive role in treatment response, as evidenced by adherence rates of 97% in the microbiologically eligible population and 91% in the safety population. Exposure-safety analysis showed that there was no association between rifampicin exposure and any grade 3 or higher AEs. In 5 participants (<1%, n = 722), we detected that increased rifampicin exposure was associated with serum total bilirubin of 3 × ULN or greater. Rifampicin is known to affect bilirubin uptake or clearance, which typically is not associated with hepatotoxicity [28–30]. Thus, this finding must be interpreted with caution, as the association is driven by a small subset of participants.

The standard dose of rifampicin is generally thought to be suboptimal and at the lower end of the dose–response curve [31, 32]. A more robust design with a range of different rifampicin doses would be required to investigate rifampicin's exposure-efficacy relationship. We acknowledge that increasing rifampicin doses has been the community's interest in shortening treatment for drug-susceptible TB. In phase 2 studies, higher doses (20, 30, and up to 35 mg/kg) were associated with improved *Mtb* elimination rates and culture conversion without increased safety concerns [6, 33–35]. RIFASHORT, a recently reported phase 3 trial, demonstrated that 4-month high-dose rifampicin regimens using flat doses of 1200 mg and 1800 mg daily did not have dose-limiting toxicities but failed to meet noninferiority criteria compared with the standard 6-month control regimen [36]. Results from the RIFASHORT experimental regimens were similar to those observed in the 4-month high-dose rifapentine without moxifloxacin regimen in S31/A5349. Together, previous phase 2 and 3 studies suggest that higher rifampicin doses improve microbiological biomarkers but not overall treatment outcomes, and that higher rifampicin doses did not appear to have dose-related toxicities. The population for the present analysis, which included 600 mg daily rifampicin as a flat dose within the context of the control arm of a large phase 3 trial, cannot be used to evaluate the risks and benefits of doses beyond 600 mg in standard 6-month regimens or shortened regimens.

Overall, covariates (sex, body weight, and race) only explained a small portion of the interindividual variability observed in rifampicin PK. Rifampicin weight-banded dosing and 600-mg flat-dosing provided comparable exposures. From simulations, a higher median AUC_ss_ in the flat-dosing strategy for the 40–50-kg weight band was not considered clinically relevant based on our exposure-efficacy and exposure-safety analyses, with minimal safety concerns while maintaining favorable long-term efficacy outcomes. Thus, flat-dosing of 600 mg daily rifampicin may be considered as an alternative to the current weight-banded dosing for adolescents and adults.

A strength of this study is its use of an unprecedentedly large rifampicin PK dataset with a robust collection of covariates. This dataset included adolescents and adults from diverse populations in 13 different countries and 34 clinical sites with varied disease burden. With PK simulation and linked exposure-efficacy and exposure-safety analyses, we provided a holistic picture of how a flat dose of 600-mg daily rifampicin regimen could have impacted clinical outcomes. Unlike previous studies, we included an array of relevant long-term efficacy and safety outcomes. We carefully considered all potential confounders that could contribute to any safety events, such as companion drug exposures and patient characteristics. We also investigated treatment efficacy via microbiological and final treatment outcomes throughout critical time points during the treatment period. Thus, we were able to offer a data-driven dosing strategy recommendation.

Our study has several limitations. First, the S31/A5349 trial did not enroll any participants below 40 kg, so we cannot infer exposures in participants with extremely low body weight. Second, we recognize that the S31/A5349 trial provided a suboptimal PK sampling schedule for rifampicin where the PK samples did not capture the expected C_max_ [22]. Since our model-derived AUC_ss_ was comparable to previous literature, we used AUC_ss_ as the exposure metric of choice in this analysis. Third, our cohort had a few patients with comorbidities (eg, HIV and diabetes), which limited our ability to infer exposures in the general TB population with certain comorbidities. Fourth, minimum inhibitory concentration (MIC) data were not available, which prevented an assessment using AUC/MIC to predict treatment outcomes. Finally, we evaluated safety outcomes in the context of combination therapy, with 2 companion drugs that are known to be hepatotoxic (isoniazid and pyrazinamide). Thus, despite adjustment for pyrazinamide exposure in multivariable analysis, our safety findings highlight associations and do not establish causal relationships for rifampicin exposure.

In conclusion, rifampicin at 600 mg daily as a flat dose yielded comparable exposure to a weight-banded dose, maintaining robust treatment efficacy with minimal safety concerns. Collectively, PK, safety, and efficacy data supported the flat-dosing strategy in the standard 6-month regimen for the treatment of drug-susceptible TB. Future research should assess the optimal dosing strategy for higher-dose rifampicin regimens.

## Supplementary Data

Supplementary materials are available at *Clinical Infectious Diseases* online. Consisting of data provided by the authors to benefit the reader, the posted materials are not copyedited and are the sole responsibility of the authors, so questions or comments should be addressed to the corresponding author.

## Supplementary Material

ciae119_Supplementary_Data

## Notes

***Author Contributions.*** E.V.K., W.C.W., E.S., K.B., W.C., M.W., M.E., K.E.D., S.E.D., P.N., S.S., R.E.C., P.N., M.L., and R.D. acquired the data and contributed to the revision of the final manuscript. H.X.N., A.Y.X., N.Z., V.K.C., and R.M.S. designed the study. H.X.N. and A.Y.X. performed the analysis and wrote the first draft of the manuscript. H.X.N., A.Y.X., G.E.V., and R.M.S. interpreted the data. All authors contributed to the intellectual content of the manuscript and approved the manuscript version submitted for publication.

***Acknowledgments.*** The authors thank all the S31/A5349 trial participants for making this work possible, and the trial networks Tuberculosis Trials Consortium (TBTC) and AIDS Clinical Trials Group (ACTG) for their support.

***Disclaimer.*** Sanofi commercial interests did not influence the study design; the collection, analysis, or interpretation of data; the preparation of this manuscript; or the decision to submit this manuscript for publication.

***Financial support*.** Sanofi (Paris, France, and Bridgewater, New Jersey, USA) donated all the study drugs in this trial, supported shipping of study drugs to all sites, and provided funding support for pharmacokinetic testing and preparation of the final Clinical Study Report in this collaborative study. H.X.N. was supported by the Univerisity of California San Francisco/Genentech Postdoctoral Fellowship in Clinical Pharmacology, and has stock in Genentech and Roche. G.E.V. was supported by the National Institute of Allergy and Infectious Diseases (NIAID) at the US National Institutes of Health (NIH) (NIH/NIAID K08 AI141740), US Agency for International Development (USAID 7200AA22CA00005), Partners In Health (SPHQ15-LOA-045), and has received consulting fees from Gates Medical Research Institute. K.E.D. was supported by NIH/NIAID (K24 AI150349). R.M.S. was supported by NIH/NIAID (R01 Al126788).

## References

[1] Iacobino A, Piccaro G, Giannoni F, Mustazzolu A, Fattorini L. Fighting tuberculosis by drugs targeting nonreplicating Mycobacterium tuberculosis bacilli. Int J Mycobacteriol 2017; 6:213–21. 28776518 10.4103/ijmy.ijmy_85_17

[2] Van Ingen J, Aarnoutse RE, Donald PR, et al Why do we use 600 mg of rifampicin in tuberculosis treatment? Clin Infect Dis 2011; 52:e194–199. 21467012 10.1093/cid/cir184

[3] World Health Organization . Companion handbook to the WHO guidelines for the programmatic management of drug-resistant tuberculosis. Geneva, Switzerland: World Health Organization, 2020. 25320836

[4] World Health Organization . WHO consolidated guidelines on tuberculosis. Module 4: treatment drug-susceptible tuberculosis treatment. Geneva, Switzerland: World Health Organization, 2022. 35727905

[5] Susanto BO, Svensson RJ, Svensson EM, Aarnoutse R, Boeree MJ, Simonsson USH. Rifampicin can be given as flat-dosing instead of weight-band dosing. Clin Infect Dis 2020; 71:3055–60. 31867594 10.1093/cid/ciz1202 PMC7819529

[6] Velásquez GE, Brooks MB, Coit JM, et al Efficacy and safety of high-dose rifampin in pulmonary tuberculosis a randomized controlled trial. Am J Respir Crit Care Med 2018; 198:657–66. 29954183 10.1164/rccm.201712-2524OC PMC6118011

[7] Sileshi T, Tadesse E, Makonnen E, Aklillu E. The impact of first-line anti-tubercular drugs’ pharmacokinetics on treatment outcome: a systematic review. Clin Pharmacol 2021; 13:1–12. 33469389 10.2147/CPAA.S289714 PMC7811439

[8] Gumbo T, Louie A, Deziel MR, et al Concentration-dependent Mycobacterium tuberculosis killing and prevention of resistance by rifampin. Antimicrob Agents Chemother 2007; 51:3781–8. 17724157 10.1128/AAC.01533-06 PMC2151424

[9] Sirgel FA, Fourie PB, Donald PR, et al The early bactericidal activities of rifampin and rifapentine in pulmonary tuberculosis. Am J Respir Crit Care Med 2005; 172:128–35. 15805182 10.1164/rccm.200411-1557OC

[10] Jindani A, Aber VR, Edwards EA, Mitchison DA. The early bactericidal activity of drugs in patients with pulmonary tuberculosis. Am Rev Respir Dis 1980; 121:939–49. 6774638 10.1164/arrd.1980.121.6.939

[11] Long MW, Snider DE Jr, Farer LS. U.S. Public health service cooperative trial of three rifampin-isoniazid regimens in treatment of pulmonary tuberculosis. Am Rev Respir Dis 1979; 119:879–94. 110184 10.1164/arrd.1979.119.6.879

[12] Pasipanodya JG, McIlleron H, Burger A, Wash PA, Smith P, Gumbo T. Serum drug concentrations predictive of pulmonary tuberculosis outcomes. J Infect Dis 2013; 208:1464–73. 23901086 10.1093/infdis/jit352 PMC3789573

[13] Ramachandran G, Agibothu Kupparam HK, Vedhachalam C, et al Factors influencing tuberculosis treatment outcome in adult patients treated with thrice-weekly regimens in India. Antimicrob Agents Chemother 2017; 61:e02464-16. 28242663 10.1128/AAC.02464-16 PMC5404592

[14] Sekaggya-Wiltshire C, Von Braun A, Lamorde M, et al Delayed sputum culture conversion in tuberculosis-human immunodeficiency virus-coinfected patients with low isoniazid and rifampicin concentrations. Clin Infect Dis 2018; 67:708–16. 29514175 10.1093/cid/ciy179 PMC6094003

[15] Dorman SE, Nahid P, Kurbatova EV, et al Four-month rifapentine regimens with or without moxifloxacin for tuberculosis. N Engl J Med 2021; 384:1705–18. 33951360 10.1056/NEJMoa2033400 PMC8282329

[16] Dorman SE, Nahid P, Kurbatova EV, et al High-dose rifapentine with or without moxifloxacin for shortening treatment of pulmonary tuberculosis: study protocol for TBTC study 31/ACTG A5349 phase 3 clinical trial. Contemp Clin Trials 2020; 90:105938. 31981713 10.1016/j.cct.2020.105938 PMC7307310

[17] R Core Team . R: a language and environment for statistical computing. Vienna, Austria: R Foundation for Statistical Computing, 2021. Available at: https://www.R-project.org/. Accessed 1 November 2022.

[18] Beal SL . Ways to fit a PK model with some data below the quantification limit. J Pharmacokinet Pharmacodyn 2001; 28:481–504. 11768292 10.1023/a:1012299115260

[19] Svensson RJ, Aarnoutse RE, Diacon AH, et al A population pharmacokinetic model incorporating saturable pharmacokinetics and autoinduction for high rifampicin doses. Clin Pharmacol Ther 2018; 103:674–83. 28653479 10.1002/cpt.778 PMC5888114

[20] Imperial MZ, Nahid P, Phillips PPJ, et al A patient-level pooled analysis of treatment-shortening regimens for drug-susceptible pulmonary tuberculosis. Nat Med 2018; 24:1708–15. 30397355 10.1038/s41591-018-0224-2 PMC6685538

[21] Therneau TM . A package for survival analysis in R 2023. Available at: https://CRAN.R-project.org/package=survival. Accessed 1 November 2022.

[22] Acocella G . Clinical pharmacokinetics of rifampicin. Clin Pharmacokinet 1978; 3:108–27. 346286 10.2165/00003088-197803020-00002

[23] Smythe W, Khandelwal A, Merle C, et al A semimechanistic pharmacokinetic-enzyme turnover model for rifampin autoinduction in adult tuberculosis patients. Antimicrob Agents Chemother 2012; 56:2091–8. 22252827 10.1128/AAC.05792-11 PMC3318330

[24] Peloquin CA, Jaresko GS, Yong CL, et al Population pharmacokinetic modeling of isoniazid, rifampin, and pyrazinamide. Antimicrob Agents Chemother 1997; 41:2670–9. 9420037 10.1128/aac.41.12.2670 PMC164187

[25] Wilkins JJ, Savic RM, Karlsson MO, et al Population pharmacokinetics of rifampin in pulmonary tuberculosis patients, including a semimechanistic model to describe variable absorption. Antimicrob Agents Chemother 2008; 52:2138–48. 18391026 10.1128/AAC.00461-07 PMC2415769

[26] Abolhassani-Chimeh R, Akkerman OW, Saktiawati AMI, et al Population pharmacokinetic modelling and limited sampling strategies for therapeutic drug monitoring of pyrazinamide in patients with Tuberculosis. Antimicrob Agents Chemother 2022; 66:e0000322. 35727060 10.1128/aac.00003-22 PMC9295566

[27] Stott KE, Pertinez H, Sturkenboom MGG, et al Pharmacokinetics of rifampicin in adult TB patients and healthy volunteers: a systematic review and meta-analysis. J Antimicrob Chemother 2018; 73:2305–13. 29701775 10.1093/jac/dky152 PMC6105874

[28] Ziakas PD, Mylonakis E. 4 months of rifampin compared with 9 months of isoniazid for the management of latent tuberculosis infection: a meta-analysis and cost-effectiveness study that focuses on compliance and liver toxicity. Clin Infect Dis 2009; 49:1883–9. 19911936 10.1086/647944

[29] Fountain FF, Tolley EA, Jacobs AR, Self TH. Rifampin hepatotoxicity associated with treatment of latent tuberculosis infection. Am J Med Sci 2009; 337:317–20. 19295414 10.1097/MAJ.0b013e31818c0134

[30] Steele MA, Burk RF, DesPrez RM. Toxic hepatitis with isoniazid and rifampin. A meta-analysis. Chest 1991; 99:465–71. 1824929 10.1378/chest.99.2.465

[31] Onorato L, Gentile V, Russo A, et al Standard versus high dose of rifampicin in the treatment of pulmonary tuberculosis: a systematic review and meta-analysis. Clin Microbiol Infect 2021; 27:830–7. 33813119 10.1016/j.cmi.2021.03.031

[32] Steingart KR, Jotblad S, Robsky K, et al Higher-dose rifampin for the treatment of pulmonary tuberculosis: a systematic review. Int J Tuberc Lung Dis 2011; 15:305–16. 21333096

[33] Aarnoutse RE, Kibiki GS, Reither K, et al Pharmacokinetics, tolerability, and bacteriological response of rifampin administered at 600, 900, and 1,200 milligrams daily in patients with pulmonary tuberculosis. Antimicrob Agents Chemother 2017; 61:e01054-17. 28827417 10.1128/AAC.01054-17 PMC5655063

[34] Svensson EM, Svensson RJ, Te Brake LHM, et al The potential for treatment shortening with higher rifampicin doses: relating drug exposure to treatment response in patients with pulmonary tuberculosis. Clin Infect Dis 2018; 67:34–41. 29917079 10.1093/cid/ciy026 PMC6005123

[35] Boeree MJ, Heinrich N, Aarnoutse R, et al High-dose rifampicin, moxifloxacin, and SQ109 for treating tuberculosis: a multi-arm, multi-stage randomised controlled trial. Lancet Infect Dis 2017; 17:39–49. 28100438 10.1016/S1473-3099(16)30274-2 PMC5159618

[36] Amina J, Daniel A, Daniel G, et al Four-month high-dose rifampicin regimens for pulmonary tuberculosis. NEJM Evid 2023; 9:EVIDoa2300054. 10.1056/EVIDoa2300054 38320155

