# Supplementary material for: Pharmacokinetic-Pharmacodynamic Evidence From a Phase 3 Trial to Support Flat-Dosing of Rifampicin for Tuberculosis
Source: Clin Infect Dis. 2024 Mar 11;78(6):1680–9. doi: 10.1093/cid/ciae119 (PMC11175687; doi:10.1093/cid/ciae119)
Supplement: ciae119_Supplementary_Data [file ciae119_supplementary_data.docx]

Supplementary Materials for Pharmacokinetic-Pharmacodynamic Evidence from a Phase 3 Trial to Support Flat-Dosing of Rifampicin for Tuberculosis

##
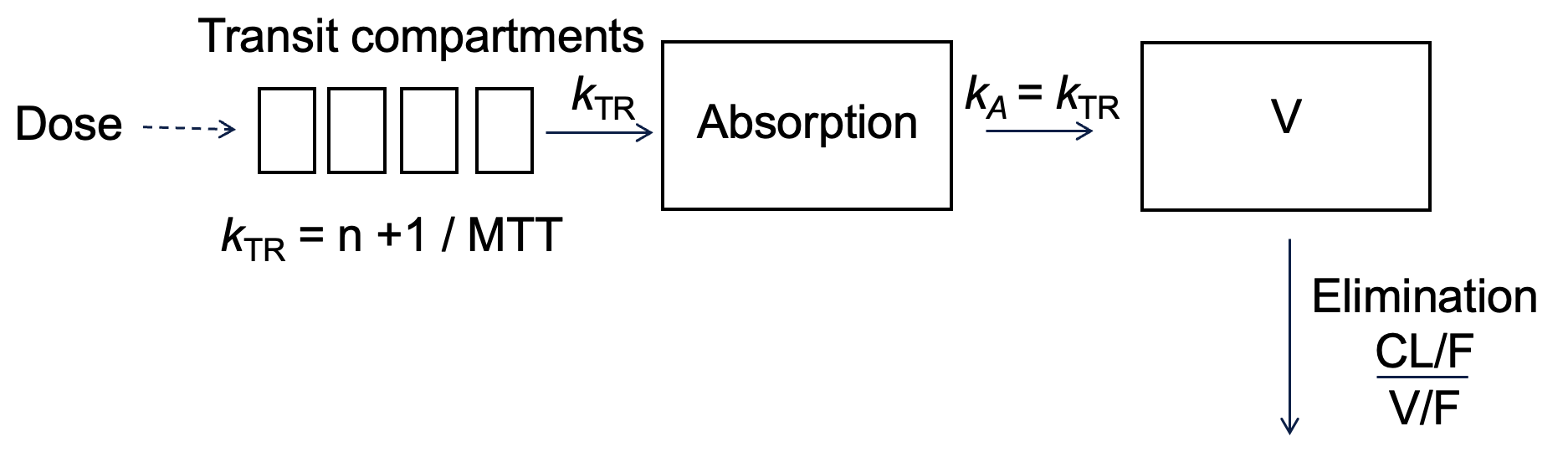


**Figure S1. Final rifampicin population PK model schematic.** Absorption compartment was characterized *via* 4 transit compartments. The transfer rate between transit compartments was defined as (n+1)/MTT. Linear clearance was used to describe the elimination process. Abbreviations: *k*_TR_ = transit rate constant; n = number of transit compartments; MTT = mean transit time; *k*_A_ = absorption rate (assumed equal to *k*_TR_); CL/F = apparent clearance; F = relative bioavailability; V/F = apparent volume of distribution.

**Table S1. Univariable and multivariable safety analysis by safety outcomes.** Safety outcomes included were significant in the univariable analysis from Table S1. Only three safety outcomes are presented, including ALT or AST ≥5xULN, serum total bilirubin ≥3xULN, and Hy’s law. ALT or AST ≥10xULN was not included due to it being a subset of the ALT or AST ≥5xULN definition. Covariates considered included participant characteristics (age, sex, body weight, height, body-mass index, race), clinical factors (cavitation, extent of disease on chest radiograph, living with HIV, diabetes, history of liver disease, smoking status) and companion drug exposures (isoniazid, pyrazinamide, ethambutol). Covariates were bolded if selected by the forward selection (P<0.10) and backward deletion method (P<0.05) using likelihood ratio test.

|  | **Univariable** | | **Multivariable** | |
| --- | --- | --- | --- | --- |
| **Predictors** | **Odds ratio**  **(95% CI)** | **P-value** | **Odds ratio**  **(95% CI)** | **P-value** |
| **ALT or AST ≥5×ULN (n/N = 15/722)** | | | | |
| Per 5 mg·L/h increase in rifampicin AUC_ss_ | 1.16 (1.07-1.26) | <0.001 | - | - |
| **Per 100 mg·L/h increase in pyrazinamide AUC_ss_** | **1.68 (1.15-2.39)** | **0.004** | **2.11 (1.41-3.10)** | **0.001** |
| Race: Asian (relative to Black) | 5.10 (1.44-17.3) | 0.008 | - | - |
| Race: White (relative to Black) | 16.9 (2.29-84.9) | 0.001 | - | - |
| **Per 10 kg increase in body weight** | **0.42 (0.18-0.89)** | **0.038** | **0.31 (0.13-0.66)** | **0.004** |
| **Serum total bilirubin ≥3×ULN (n/N = 5/722)** | | | | |
| **Per 5 mg·L/h increase in rifampicin AUC_ss_** | **1.36 (1.18-1.64)** | **<0.001** | **1.36 (1.17-1.65)** | **<0.001** |
| Per 100 mg·L/h increase in pyrazinamide AUC_ss_ | 2.17 (1.31-3.62) | 0.001 | - | - |
| **Per 10 years increase in age** | **2.81 (1.46-6.01)** | **0.003** | **2.51 (1.28-5.82)** | **0.014** |
| Race: White (relative to Black) | 23.3 (1.04-261) | 0.013 | - | - |
| History of liver disease | 35.6 (1.67-304) | 0.003 | - | - |
| **Hy’s law**^a^ **(n/N = 4/722)** | | | | |
| **Per 5 mg·L/h increase in rifampicin AUC_ss_** | **1.25 (1.07-1.46)** | **0.004** | **1.23 (1.05-1.45)** | **0.0094** |
| Per 100 mg·L/h increase in pyrazinamide AUC_ss_ | 1.97 (1.08-3.31) | 0.009 | - | - |
| **Per 10 years increase in age** | **2.88 (1.38-6.76)** | **0.007** | **2.53 (1.22-6.17)** | **0.020** |
| Race: White (relative to Black) | 23.3 (1.04-261) | 0.013 | - | - |
| History of liver disease | 47.5 (2.15-457) | 0.002 | - | - |

Abbreviations: AUC_ss_ = area under the plasma concentration-time curve at steady state; OR = odds ratio; CI = confidence interval; n/N = number of participants reported for each safety outcome out of the total number of participants analyzed; AE = adverse event; ALT = alanine transaminase; AST = aspartate transaminase; ULN = upper limit of normal.

^a^Hy’s law is defined as ALT or AST level ≥3×ULN plus total bilirubin level ≥2×ULN.

**Table S2. Odds of safety outcomes by rifampicin exposure.** Odds ratios were calculated per 5 mg·h/L increase in rifampicin AUC_ss_ after adjustment for pyrazinamide AUC_ss_, age, and body weight. Safety and tolerability outcomes were defined as those occurring during treatment and up to 14 days after discontinuation of study drug. Safety outcomes significantly associated with rifampicin in both univariable and multivariable analyses are bolded.

|  | **Event rate**  **(%, n/total N)** | **Unadjusted OR**  **(95% CI)** | **Unadjusted p-value** | **Adjusted OR**  **(95% CI)** | **Adjusted**  **p-value** |
| --- | --- | --- | --- | --- | --- |
| **Total safety population** | | | | | |
| Primary safety outcome:  Grade ≥3 adverse event | 17.87 (129/722) | 1.02 (0.98-1.06) | 0.3 | 1.01 (0.96-1.06) | 0.6 |
| Secondary safety outcome: Grade ≥3 treatment-related adverse event | 9.70 (70/722) | 1.05 (0.99-1.10) | 0.08 | 1.02 (0.96-1.08) | 0.5 |
| Discontinuation of assigned treatment due to any adverse event | 0.28 (2/722) | 1.02 (0.70-1.29) | 0.9 | 0.67 (0.30-1.00) | 0.2 |
| Grade ≥3 adverse event within 28 weeks post-randomization | 17.87 (129/722) | 1.02 (0.98-1.06) | 0.3 | 1.01 (0.96-1.06) | 0.6 |
| **Serum total bilirubin ≥3×ULN** | **0.69 (5/722)** | **1.36 (1.18-1.64)** | **<0.001** | **1.37 (1.11-1.77)** | **0.006** |
| ALT or AST ≥5×ULN | 2.08 (15/722) | 1.16 (1.07-1.26) | <0.001 | 1.07 (0.96-1.19) | 0.2 |
| ALT or AST ≥10×ULN | 0.69 (5/722) | 1.24 (1.09-1.43) | 0.001 | 1.08 (0.90-1.31) | 0.4 |
| Serious adverse event | 5.54 (40/722) | 1.06 (0.99-1.12) | 0.07 | 1.01 (0.93-1.09) | 0.8 |
| Hy’s law^a^ | 0.55 (4/722) | 1.25 (1.07-1.46) | 0.004 | 1.19 (0.95-1.48) | 0.1 |
| Death | 0.55 (4/722) | 1.13 (0.94-1.31) | 0.1 | 1.16 (0.93-1.39) | 0.1 |
| **Tolerability (Microbiologically eligible population)** | | | | | |
| Discontinuation of assigned regimen for any reason | 5.29 (36/680) | 0.96 (0.88-1.04) | 0.3 | 0.93 (0.84-1.02) | 0.1 |

Abbreviations: AUC_ss_ = area under the plasma concentration-time curve at steady state; OR = odds ratio; CI = confidence interval; AE = adverse event; ALT = alanine transaminase; AST = aspartate transaminase; ULN = upper limit of normal.

^a^Hy’s law is defined as ALT or AST level ≥3×ULN plus serum total bilirubin level ≥2×ULN.
